# Mizoribine: A New Approach in the Treatment of Renal Disease

**DOI:** 10.1155/2009/681482

**Published:** 2009-12-13

**Authors:** Yukihiko Kawasaki

**Affiliations:** Department of Pediatrics, Fukushima Medical University School of Medicine, 1 Hikariga-oka, Fukushima City, Fukushima 960-1295, Japan

## Abstract

Mizoribine (MZB) is an imidazole nucleoside and an immunosuppressive agent. The immunosuppressive effect of MZB has been reported to be due to the inhibition of DNA synthesis in the S phase of the cell cycle. Because of its relative lack of toxicity, during the past decade MZB has been frequently used instead of azathioprine as a component of immunosuppressive drug regimens. MZB is being used to treat renal transplantation patients, IgA nephropathy, lupus erythematosus, and childhood nephrotic syndrome (NS), and some recent studies have assessed the efficacy of oral MZB pulse therapy for severe lupus nephritis, steroid-resistant NS, and frequently relapsing-steroid-dependent NS. This review summarizes the published findings on the efficacy of MZB for renal disease including IgA nephropathy, lupus nephritis, and NS, as well as of oral MZB pulse therapy for severe lupus nephritis and NS, and also the mechanism of the effect of oral MZB pulse therapy on the lymphocyte cell cycle.

## 1. Introduction

*Eupenicillium brefeldianum*, an ascomycetes harvested from the soil of Hachijo Island, Tokyo, Japan, in 1971, produces mizoribine (MZB). MZB is a nucleoside of the imidazole class, and was found to have weak antimicrobial activity against *Candida albicans*, but it proved ineffective against experimental candidiasis [1].

 MZB inhibits the de novo purine biosynthesis of purines, but unlike azathioprine (AZT), it is not incorporated into nucleic acids in the cell. Instead, after phosphorylation, misoribine-5′-monophosphate (MZB-5′P) inhibits guanosine monophosphate (GMP) synthesis by antagonistic blocking of inosine monophasphate dehydrogenase (IMPDH) and GMP-synthetase in the pathway from inosine [5′-] monophasphate (IMP) to GMP in the purine synthesis system. MZB was found to inhibit both humoral and cellular immunity by selectively inhibiting lymphocyte proliferation, which led to its development as an immunosuppressive agent. The clinical efficacy of MZB as an immunosuppressant for renal transplantation was investigated in various Japanese institutions during the period from 1978 to 1982, and in 1984, MZB was approved by the Japanese Ministry of Health, Labour and Welfare as a drug indicated for the prevention of rejection in renal transplantation [2, 3]. Recently, it has been most commonly used in combination with other immunosuppressants, such as cyclosporine (CyA) or tacrolimus, and corticosteroids, for transplantation.

 The characteristics of MZB, which differentiate it from AZT, are the lack oncogenicity shown in animal experiments and association with a low incidence of severe adverse drug reactions, for example, myelosuppression and hepatotoxicity, clinically [1–3]. Since these findings suggested that MZB would be useful for long-term immunosuppressive therapy, several clinical trials of MZB for the treatment of autoimmune diseases were carried out, and its clinical usefulness was obvious. In addition to its approval for the prevention of rejection after renal transplantation, MZB has been approved in Japan for the treatment of lupus nephritis (1990), rheumatoid arthritis (1992), and primary nephritic syndrome (1995), and in these diseases, it has often been used in combination with corticosteroids and/or anti-inflammatory drugs [4]. 

Nevertheless, because of its relatively low-efficacy MZB is still not widely used clinically. It is one of the causes that blood concentration of MZB does not increase enough. The peak blood levels of MZB during standard MZB therapy, that is, 3 mg/kg daily in three divided dose, has been reported to be approximately 0.5 *μ*g/mL [5], lower than the concentration required to inhibit experimental human MLRs, which occurs in the 3.0–6.0 *μ*g/mL concentration range [6]. To increase the its peak blood levels, MZB was has recently been administrated in a single daily dose of 150 mg or at a total daily dose of 6–10 mg/kg in a single dose or two divided doses, twice a week, and has been reported to be effective. 

This review summarizes the mechanism of action of MZB and the published findings on the efficacy of MZB for renal disease including IgA nephropathy, lupus nephritis, and NS, and on the efficacy of oral MZB pulse therapy for severe lupus nephritis and NS, and on the mechanism of the effect of oral MZB pulse therapy on the lymphocyte cell cycle.

## 2. Mechanism of Action of MZB

 MZB has a very specific mechanism of action on the lymphocytes that inhibits their proliferation without interfering with purine synthesis in other cell types. Purine synthesis occurs via two separate pathways: a de novo pathway and a salvage pathway. In the de novo pathway, the ribose phosphate portion of purine nucleotides is derived from 5-phosphoribosyl 1-pyrophosphate (PRPP), which is synthesized from ATP and ribose 5-phosphate, and lymphocytes are primarily dependent on the de novo pathway [7–9]. In the salvage pathway, purine bases, sugars, and other products are essentially recycled, and most cells, including polymorphonuclear leukocytes and neurons, are able to utilize the salvage pathway. The specificity of the inhibitory effect of MZB on lymphocytes proliferation is attributable to the fact that it acts on the de novo pathway, of purine biosynthesis alone and does not act on the salvage pathway in purine biosynthesis.

Sakaguchi et al. [10, 11] investigated the mechanism of action of MZB in mouse lymphoma cell line L5178Y, which is very sensitive to MZB, and found that MZB strongly inhibited both DNA synthesis and RNA synthesis, but not protein synthesis. AZT, which also exerts its immunosuppressive effect through antimetabolism, is known to be incorporated into nucleic acid instead of thioguanosine 5′-triphosphate [12]. MZB, however, was found not to be incorporated into DNA or RNA in a study using [^14^C] MZB, but did specifically inhibit nucleic acid synthesis [13]. MZB almost completely suppresses the growth of L5178Y cells at a concentration of 10^−5^ M. The addition of 2 × 10^−4^ M GMP to this culture system liberates the cells from growth inhibition by MZB, but other purine nucleotides or pyrimidine nucleotides do not reverse the effect of MZB [13]. Based on these findings, MZB can be concluded to inhibits the synthesis of GMP from IMP in the purine metabolism pathway, without being incorporated into DNA or RNA. 

Koyama and Tsuji [14] demonstrated that MZB is metabolized into an active form, MZB 5′-P, by adenosine kinase (AK) in carcinoma cells. They used cells that were resistant to various drugs and had been obtained by treating mouse mammary carcinoma cells (FM3A) with N-methyl-N′-nitro-N-nitrosoguanidine. The MZB-resistant (MZB^r^) mutants that were obtained as a result were 15–19-fold less sensitive to MZB than wild-type cells. The MZB^r^ mutants were capable of incorporating radioactivity from ring-labeled adenosine into their acid-insoluble macromolecular fraction the same as wild-type cells, but hypoxanthine-guanine phosphor-ribosyl-transferase deficient (HGPRT^T-^) mutants derived from the MZB^r^ cells did not incorporate the radioactivity at all or incorporated it at a much lower rate. Exogenous adenosine enters the purine nucleotide pool via two different pathway. In one pathway, it is phosphorylated by AK and then metabolized to adenosine 5′-monophosphate (AMP), whereas in the other, pathway it is converted to inosine through the action of adenosine deaminase, and the inosine is then converted to hypoxanthine (by purine nucleoside phosphorylase) and is metabolized into IMP (by HGPRT). The two pathways of adenosine metabolism described above are blocked. Enzyme assays of cell-free extracts of the MZB^r^ mutants revealed that their AK activity was less than 3% of the AK activity found in wild-type cells. Based on these findings, it was clear that MZB suppressed cell growth in the presence of AK, strongly suggesting that MZB exerts its suppressive effect on cell growth only after being metabolized to MZB-5′P by AK.

Kusumi et al. [15] investigated the inhibitory effects of MZB and MZB-5′P using cell-free extracts from rat liver on IMPDH and Walker sarcoma cells on GMP synthetase. MZB inhibited neither enzyme, whereas MZB-5′P inhibited both, and its Ki values were 10^−8^ M IMPDH and 10^−5^ M for GMP synthetase. These results demonstrated that the suppressive effect of MZB on cell growth is attributable to MZB-5′P and not to MZB itself, and that MZB-5′P primarily inhibits IMPDH, and secondarily inhibits GMP synthetase, thereby inhibiting two enzymes that act in two sequential steps in the GMP synthesis process. MZB-5′P appears to almost completely inhibits guanine nucleotide synthesis. Quantitative changes in purine nucleotides in MZB-treated cells have also been investigated to confirm the enzyme-inhibiting effect of MZN-5′P. However, L5178Y cells, in which de novo purine nucleotide synthesis had been arrested with aminopterin, were incubated with ^14^C-labeled hypoxanthine, in the presence or absence of MZB. When the purine nucleotides were isolated, and the radioactivity in each of the nucleotides was measured, the amount of GMP-containing guanine nucleotide was found to have decreased considerably after incubation in the presence of MZB, in comparison to incubation in the absence of MZB.

 Turka et al. [16, 17] investigated the effect of MZB on human peripheral blood cells stimulated with anti-CD3 monoclonal antibodies or pharmacological mitogens and found that MZB inhibited T cell proliferation by 10–100% in a dose-dependent manner in relation to all stimuli tested. MZB also caused a dose dependent decrease in GTP pools, and addition of guanosine both prevented the GTP depletion and reversed its antiproliferative effect at all but the highest doses of MZB. 

### 2.1. In Vitro Effects

#### 2.1.1. Growth Inhibitory Effects of MZB on Various Cells

Mizuno et al. investigated the growth inhibitory effect of MZB on several cell lines and showed that MZB had a strong inhibitory effect on lymphoma cell line L5178Y and L-cells, with IC50 values >100 [1].

#### 2.1.2. Effect of MZB on Lymphocytes Stimulated with Mitogens or Allogenic Cells

 Kamata et al. [2] studied the effect of MZB on lymphocytes from beagle dogs and observed dose-dependent inhibition of the blastogenic response of lymphocytes to concanavalin A, phytohemagglutinin, and pokeweed mitogen, as well as of the mixed lymphocyte reaction (MLR).

 Ichikawa et al. [3] investigated the effect of MZB on proliferation by human lymphocytes and showed that MZB suppressesd their blastogenic response to all three of the above mitogens, and the MLR. The mitogen responses and MLR were significantly suppressed at a concentration of 10 *μ*g MZB/mL, and the 50% inhibition doses of MZB against the three mitogens and on the MLR were between 1.0 and 10 *μ*g/mL.

#### 2.1.3. Novel Mechanism of Action of MZB

Itoh et al. [18] used MZB affinity column chromatography and porcine kidney cytosols to identify proteins that specifically bind MZB using MZB affinity column chromatography and porcine kidney cytosols. By increasing MZB in the eluant from the column, two major proteins (with molecular masses of 60 and 43 kDa) were detected by sodium dodecyl sulfate- (SDS-) polyacrylamide gel electrophoresis. Based on the amino acid sequence analysis of these proteins, 60- and 43-kDa MZB-binding proteins were identified with heat shock protein (HSP) 60 and cytosolic actin, respectively. A considerable amount of actin was also eluted from the affinity column by nucleotides, but a very low quantity of HSP60 was eluted under the same conditions. On the other hand, HSP60 was eluted as a major protein in the eluant that was eluted preferentially, with nucleotide followed by MZB. Actin was also detected in the eluant, but the quantity of the protein was very low. These results indicated that HSP60 had a high affinity to MZB, and the interaction was also observed on surface plasmon resonance analysis. 

The 14-3-3 proteins form a highly conserved family of acidic, dimeric proteins that are widely distributed among eukaryotic cells. The 14-3-3 proteins interact with many proteins involved in cellular signaling, including the glucocorticoid receptor (GR), and the 14-3-3/GR interaction enhances the transcriptional activity of the receptor. Takahashi et al. showed that MZB affected the conformation of 14-3-3 proteins and enhanced the interaction between GR and 14-3-3 dose-dependently in vitro. MZB also has a stimulatory effect on transcriptional activation by the GR. These findings point to the possibility that regulation of the GR function via 14-3-3 proteins may be one of the mechanisms of the therapeutic effect of MZB [19].

#### 2.1.4. Absorption, Distribution, Metabolism, and Excretion of MZB

 The absorption, distribution, metabolism, and excretion of MZB were investigated after orally administering ^14^C-MZB to rats [20], MZB was rapidly absorbed, and its blood concentrations peaked at 1.5 hours, then rapidly declined. MZB was almost completely eliminated within 24 hours. Whole-body autoradiography revealed high levels of radioactivity in the stomach, small intestine, liver, kidney, spleen, and thymus one hour after administration. Within 24 hours, 85% of the administered dose was excreted in the urine and 1.0% in the bile. Inverse isotope dilution analysis showed that unchanged ^14^C-MZB accounted for more than 99% of the radioactivity in the plasma one hour after dosing, and the 85% of MZB excreted in the urine within 24 hours after administration was unchanged.

 When a 100 mg oral dose of MZB was administrated to six kidney-transplantrecipients with good renal function, and a serum creatinine levels under 2.7 mg/dL, their serum MZB concentration peaked at about 2.3 g/mL two hours after the dose, then gradually decreased T_1/2_ value was 2.2 hours. About 82% of the oral dose of MZB had been excreted in the urine of the transplant patients six hours after administration [21]. The serum MZB concentration of patients with renal dysfunction remained high even 24 hours after administration. The rate of MZB elimination from serum is closely correlated with renal function [22].

#### 2.1.5. Blood MZB Levels and Oral MZB Pulse Therapy

**Figure 1:**
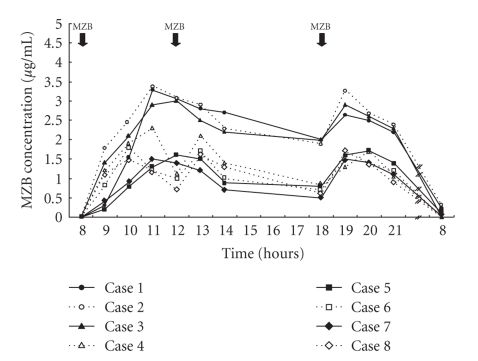
Change in the serum MZB concentration of each patient on the days when MZB was administered.

The peak blood level of MZB, during regular MZB therapy, that is, 3 mg/kg daily in three divided, the peak levels of the drug has been reported to be approximately 0.5 *μ*g/mL [5]. It has recently been reported that peak blood MZB levels in the 3.0–6.0 *μ*g/dL are sufficient to inhibit the human MLR [6]. Thus, the higher serum MZB concentrations achieved by pulse therapy are needed to inhibit disease activity. Stypinski et al. [23] reported that higher doses than the current clinical dosage of 2–5 mg/kg day may be needed to maintain the efficacy of MZB. The safety, tolerability and pharmacokinetics of MZB in two clinical trials of higher-dose MZB administration to healthy male volunteers have been reported. Forty-eight healthy White male nonsmokers participated in two randomized, double-blind, placebo-controlled trials: 32 in a single-dose study (3, 6, 9, and 12 mg/kg) and 16 in a multiple-dose study (6 mg/kg/day once daily for 5 days or twice daily for 7 days), and standard assessments of safety, tolerability, and pharmacokinetics were performed. The safety profiles in both studies were generally unremarkable, except for elevated serum uric acid concentrations at the highest dose (12 mg/kg/day) in the multiple-dose study. After oral MZB reached its peak serum concentrations within 2-3 hours and was eliminated mostly via the kidney (65–100% of dose), its serum half-life was 3 hours. Only the 12 mg/kg/day group had trough concentrations that were within the therapeutic window (trough concentrations >0.5 but <3 *μ*g/mL). Based on the safety profile of MZB and current pharmacokinetic information, a new starting dose in the 6–12 mg/kg/day range is recommended for kidney transplant patients in the up to 3-month acute phase following transplantation. Kawasaki et al. reported a peak serum MZB concentration of 1.4–4.8 *μ*g/mL and a morning trough serum MZB concentration of 0–0.3 in 8 patients with NS when MZB was given orally in a dose of 10 mg/kg body weight daily (maximum total daily dose 500 mg) in three divided doses, 2 days a week (Figure 1) [24, 25]. In addition, Kawasaki et al. found that the peak serum concentration was 3.0–5.1 *μ*g/mL, the AUC 0–4 of MZB was 7.0–16.0 *μ*g · h/mL and the morning trough serum MZB concentration was 0 *μ*g/mL (Table 1), when MZB was given orally in a dose of 6 mg/kg body weight daily (maximum total dose 300 mg) twice a week in 11 patients with frequently relapsing NS [26]. 

## 3. Clinical Efficacy

### 3.1. Renal Transplantation

The clinical efficacy of MZB as an immunosuppressant for renal transplantation was assessed in various Japanese institutions during the period between 1978 and 1982, the period when immunosuppression was mainly achieved with AZT and corticosteroids and before the immunophilin-binding drugs cyclosporine (CyA) or tacrolimus were available. During that period, 200 to 300 renal transplants were performed in Japan each year. In one study the immunosuppressive effect of triple-drug therapy (MZB + AZT + corticosteroid) in 57 cases was compared with the immunosuppressive effect obtained in 72 historical controls treated with AZT + corticosteroid alone. The graft survival rate in the group that received MZB, was 89.6%, and significantly higher (*P* < .05) than the (74.6%) in the group that did not receive MZB [4].

From 1989 through 1998, Tanabe et al. [27] executed a prospective, randomized study to evaluate the immunosuppressive effect of MZB in 116 renal transplantation patients. Patients received MZB or AZT for 9 years after transplantation. The 9-year patient survival rate of the MZB group and AZT group was 88% and 83%, respectively. The 9-year graft survival rates of the MZB group was 58% and 52%, respectively, and differences between the groups in graft survival rate and patient survival rate were not significant. However, AZT had to be switched to MZB in 16 patients (27.6%) because of adverse effects, which consisted of myelosuppression in 11 patients and liver dysfunction in 5 patients. No MZB-related adverse effects occurred, and discontinuation of MZB was never necessary. According to these results, MZB has almost the same immunosuppressive effect as AZT but many fewer adverse effects.

### 3.2. IgA Nephropathy (IgAN)

Primary immunoglobulin A (IgA) nephropathy (IgAN) is a disease that was first reported in 1968 by Berger and Hinglais and is characterized by microhematuria and proteinuria clinically, and by deposition of IgA histologically. IgAN is the most common form of chronic glomerulonephritis worldwide, and in up to 30% of patients it progresses to end-stage renal failure. Since severe IgAN could not be controlled with a single drug, combinations of drugs with different mechanism of action, including corticosteroids, immunosuppressive agents, antiplatelet drugs, and anticoagulation, have been used. The rationale for using prednisolone and MZB in IgAN is that corticosteroids and immunosuppressive agents reduce IgA production and minimize the abnormal immune response and inflammatory events following glomerular IgA deposition. Warfarin and dilazep dihydrochloride are used to inhibit the mediators of glomerular damage.

 Kaneko et al. [28] showed that MZB was effective against moderately severe childhood IgAN because of its antiproteinuric effect and lower toxicity. Nagaoka et al. [29] further found that MZB could be used as an alternative drug to treat moderately severe childhood IgAN because MZB resulted in a significant reduction of proteinuria and hematuria with histological improvement and caused far fewer complications than the conventional immunosuppressants. To evaluate the efficacy of prednisolone, warfarin, dilazep dihydrochloride combined with MZB (multiple drug combination therapy (PWDM)) for diffuse IgAN in childhood, Kawasaki et al. retrospectively compared the clinical features and pathology findings of diffuse IgAN patients treated with PWDM with those of patients who received multiple-drug therapy without MZB (PWD) and multiple-drug therapy in combination with methylprednisolone pulse therapy (PWD-pulse) (Tables 2, 3, and 4). The duration of follow-up (years) was 8.9 ± 5.2 in the PWD group, 8.1 ± 3.9 in the PWD-pulse group, and 7.7 ± 3.8 in the PWDM group. At the most recent follow-up examination, mean urinary protein excretion (mg/m^2^/h) was 17 ± 10 in the PWD group, 22 ± 20 in the PWD-pulse group, and 6 ± 6 in the PWDM group, and had decreased significantly in the PWDM group in comparison with the other groups. The activity index (AI) in all three groups was lower at the second biopsy than that at the first biopsy (5.1 ± 0.8 versus 6.5 ± 2.1 in PWD group, *P* < .05; 5.6 ± 0.9 versus 6.6 ± 1.7 in PWD-pulse group, *P* < .01; and 4.5 ± 1.0 versus 6.8 ± 1.9 in the PWDM group, *P* < .01). The chronicity index (CI) in the PWD group and PWD-pulse group at the second biopsy was higher than at the first biopsy (7.3 ± 1.4 versus 4.8 ± 1.0 in the PWD group, *P* < .01; 8.1 ± 2.0 versus 5.3 ± 0.9 in the PWD-pulse group, *P* < .01), but was unchanged in the PWDM group. At the most recent follow-up examination, two patient (10%) in the PWD group, 3 (15%) in the PWD-pulse group, and 12 (60%) in the PWDM group had renal insufficieny, 1 patient (4.8%) in the PWD group, 3 (15%) in the PWD-pulse group, and none (0%) in the PWDM group had normal urine, 7 patient (35%) in the PWD group, 6 (30%) in the PWD-pulse group, and 7 (35%) in the PWDM group had minimal urinary abnormalities; while 11 patient (52%) in the PWD group, 8 (40%) in the PWD-pulse group, and 1 (5%) in the PWDM group had persistent nephropathy; finally, 1 patient (5%) in the PWD group, 3 (15%) in the PWD-pulse group, and none (0%) in the PWDM group had renal insufficiency. These results suggest that PWDM is more effective than PWD or PWD-pulse in reducing the proteinuria and histological severity in patients with IgAN. In addition, we prospectively investigated the efficacy of PWDM against IgAN. After 6 months of treatment mean urinary protein excretion had decreased significantly compared to before the start of treatment [30]. The incidence of hematuria after PWDM therapy was lower than that before the start of treatment. The AI decreased significantly from 4.8 ± 2.1 at the first biopsy to 2.3 ± 1.7 at the second biopsy (*P* < .001) and the CI decreased significantly from 4.1 ± 1.9 at the first biopsy to 2.7 ± 2.4 at the second biopsy (*P* < .05). Macrophage infiltration and alpha-smooth muscle actin positive cells in the glomerulus and interstitial region decreased significantly between before therapy and after therapy, and the serum IgA levels (mg/dL) was lower after therapy (197.4 ± 78.1 versus 266.5 ± 105.0, *P* < .01, resp.). At the most recent follow-up examination, none of the 34 patients had renal insufficiency. These findings suggested that the prednisolone plus MZB combination therapy is effective in patients who are at risk of progression of IgAN. The rationale for using prednisolone and MZB in IgAN is that corticosteroids and immunosuppressive agents reduce IgA production and minimize the abnormal immune response and inflammatory events following glomerular IgA deposition. Warfarin and dilazep dihydrochloride are used to inhibit the mediators of glomerular damage. Most of the side effects were mild and well controlled, and all were reversible. Severe side effects attributable to the prednisolone plus MZB combination therapy regimen were relatively rare and the regimen was well tolerated and safe in all patients.

Yoshikawa et al. [31] treated 23 children with severe IgAN with MZB, prednisolone, heparin-warfarin, and dipyridamole, and evaluated their efficacy and safety. The primary endpoint, a urine protein/creatinine ratio <0.2, was achieved in 18 patients during the two-year treatment period. The cumulative proteinuria resolution rate determined by the Kaplan-Meier method was 80.4%, and median protein excretion decreased from 1.19 g/m^2^/day to 0.05 g/m^2^/day (*P* < .0001). The median percentage of glomeruli showing sclerosis was unchanged in comparison with before treatment. No patients required a change treatment. In conclusion, the efficacy and safety of the MZB combination seem acceptable for treating children with severe IgAN.

### 3.3. Nephrotic Syndrome

During the period between 1989 and 1991, Koshikawa et al. [32] performed a 24-week, prospective, randomized, double-blind, placebo-controlled, comparative trial to assess the efficacy of MZB in patients with steroid-resistant NS, and the efficacy was assessed in a total of 158 patients (80 in the MZB group and 78 in the placebo group). The global improvement rate, evaluated by the physicians in charge, was significantly higher in the MZB group (33.8%) than in the placebo (14.1%) group (*P* < .05), and the difference between the improvement rates in the two groups became more marked when the subgroup taking corticosteroids at daily doses below 20 mg as a prednisolone rquivalent at baseline (30.0% versus 5.3%, *P* < .05) was evaluated. Laboratory studies revealed an average 25.2% reduction in the urinary protein level in the MZB group as opposed to 10.0% in the placebo group (*P* < .0). The incidences of side effects in the MZB group (13.6%) and placebo groups (11.9%) did not differ significantly.

Yoshioka et al. [33] showed that MZB significantly decreased the relapse rate and prolonged remission in a subgroup of NS patients <10 years old, and that it can be useful in young children, who generally have a higher relapse rate than older children.

 Kawasaki et al. [25] evaluated the efficacy of oral MZB pulse therapy (10 mg/kg body weight daily (maximum total daily dose 500 mg) in three divided doses, 2 days a week) in one child with cyclosporine-dependent steroid-resistant NS and eight children with frequently relapsing steroid-dependent NS, and found that four patients had no subsequent relapses (responders). Prednisolone and CyA were discontinued in two of the four responders, and CyA was discontinued in the other two. Although each of the five other patients (non-responders) experienced a single subsequent relapse, after MZB pulse therapy the dosages of prednisolone and CyA were significantly reduced in comparison with before MZB pulse therapy. The peak blood concentration of MZB in the responders was higher than in the nonresponders (3.6 ± 0.9 versus 1.8 ± 0.4 *μ*g/mL, *P* < .05).

Kawasaki et al. [26] on the other hand, demonstrated the efficacy of single dose oral MZB pulse therapy (6 mg/kg body weight daily (maximum total daily dose 300 mg) twice a week) in 11 patients with frequently relapsing steroid-dependent NS and found that eight of the 11 had no subsequent relapses (responders), and prednisolone could be discontinued [25]. Although 2 of the other 3 patients (nonresponders) had one relapse and the remaining patient had two relapses, the dosage of prednisolone and frequency of relapse after oral MZB pulse therapy were significantly lower than before oral MZB pulse therapy. The peak blood concentration and AUC 0–4 of MZB in the responders were higher than in the nonresponders. None of patients had severe adverse effects, such as uricacidemia, leucopenia, liver dysfunction, or alopecia. 

In addition, Ohtomo et al. [34] showed that high-dose MZB therapy appeared to be effective in reducing cyclosporine exposure as well as in decreasing the frequency of relapses in patients with frequently relapsing steroid-dependent NS who are also cyclosporine-dependent. Thus, these findings suggested that oral MZB pulse therapy may be effective in some patients with cyclosporine-dependent steroid-resistant NS and frequently relapsing steroid-dependent NS.

### 3.4. Lupus Nephritis

Yumura et al. [35] investigated whether maintenance therapy with MZB and prednisolone could improve immunity, reduce proteinuria, prevent renal relapse, and allow reduction of the steroid dose in severe proliferative lupus nephritis patients. Long-term maintenance therapy with MZB and prednisolone was evaluated in ten patients with biopsy-proven severe proliferative lupus nephritis, and 0.5 g or more proteinuria even after treatment by plasma exchange and/or with pulse methylprednisolone. MZB at an average dose of 140 ± 10 (100–200) mg was administered 2–3 times daily/day in combination with prednisolone. The average duration of MZB maintenance therapy was 89.7 ± 5.5 (70–126) months. All patients were females, and their mean age was 43.0 ± 3.3 years. A significant decrease in proteinuria was noted two years after the start of combination therapy (*P* = .0016). The serum creatinine levels of all patients remained unchanged throughout the treatment and follow-up period, even during renal relapses. The C3 and CH50 levels became normal as the proteinuria decreased. None of the patients developed serious side effects during MZB treatment. A significant steroid-sparing effect was observed three years after the start of MZB therapy (*P* = .0025). Based on the results of long-term follow-up, maintenance therapy with low-dose prednisolone combined with MZB can eliminate proteinuria and has a steroid-sparing effect. Early initiation of therapy can protect patients with severe proliferative lupus nephritis against renal relapses without serious side effects.

Tanaka et al. [36] assessed oral MZB pulse therapy for lupus nephritis and reported that oral MZB pulse therapy was effective in five patients with a long history of systemic lupus erythematosus (SLE), including four patients with proliferative lupus nephritis (WHO class IV) and one patient with WHO class II lupus nephritis, in whom remission had been achieved by treatment with high-dose corticosteroids combined with cytotoxic agents. The patients were treated with MZB 5–10 mg/kg daily (up to 500 mg daily) in a single daily dose two days a week (Monday and Thursday) for over 24 months. The dose of the concomitant corticosteroid was gradually reduced or continued unchanged. On presentation, the urinary protein excretion, serum complement hemolytic activity (CH50) and serum anti-dsDNA antibody titer were 1.7 ± 1.0 g/day, 16.6 ± 3.8 U/mL (normal, 23–46 U/mL) and 143.7 ± 151.1 IU/mL (normal, <12.0 IU/mL), respectively. At the most recent follow-up examination, after a mean interval of 31 months (24–34 months) since the start of MZB pulse therapy, the urinary protein excretion and serum anti-dsDNA antibody titer had significantly decreased (0.3 ± 0.2 g/day and 18.5 ± 19.1 IU/mL, resp.; *P* < .05), and the serum CH50 value had returned to within normal range (33.6 ± 7.8 U/mL, *P* < .05). Despite the reduced minimum dose of prednisolone required to maintain clinical remission at the time of the post-treatment evaluation after MZB pulse therapy as compared with that at the time of the pretreatment evaluation (9.0 ± 4.5 versus 17.5 ± 7.9 mg/day; *P* = .0656), the calculated flare rate significantly decreased (0.15 ± 0.2 versus 0.6 ± 0.11 times per year; *P* < .05). The serum creatinine level remained within the normal range in all the participants in the study, and the platelet count of two patients with chronic thrombocytopenia increased following the MZB pulse therapy. No serious adverse effects were observed. These findings suggest that long-term MZB pulse therapy may be the treatment of choice for selected lupus nephritis patients who are at high risk of relapse. Futhermore, Tanaka et al. [37] reported on the efficacy of the oral MZB pulse protocol for induction therapy for newly diagnosed childhood-onset systemic lupus erythematosus (SLE). Five consecutive newly diagnosed SLE patients with biopsy-proven lupus nephritis were recruited for an open-label trial of prednisolone and MZB intermittent pulse therapy (10 mg/kg 2 days a week for 12 months). Data on the renal response and serologic lupus activity were collected prospectively. The baseline characteristics of the patients were: mean age, 11 years; urinary protein/creatinine ratio (U-prot./cre.), 0.99 ± 0.91; serum complement hemolytic activity (CH50), 10.6 ± 1.3 (normal, 23–46 U/mL); serum anti-dsDNA antibody titer, 258.6 ± 125.5 IU/mL (normal, <12.0 IU/mL); serum creatinine, 0.5 ± 0.1 mg/dL; and European Consensus Lupus Activity Measurement index (ECLAM), 7.4 ± 1.1. The primary endpoint was the interval until the development of a flareup of SLE. Despite gradual tapering of the prednisolone dose, significant improvement in all parameters examined was observed at 3, 6, and 12 months of treatment in comparison with the baseline values. After 12 months of therapy, a complete response was achieved in all of the patients, except 1 patient with poor drug compliance. Marked histological improvement was confirmed at the second renal biopsy in two patients found to have severe lupus nephritis at the first renal biopsy. No serious adverse effects were observed. Thus, the MZB pulse protocol combined with prednisolone for induction therapy may be the treatment of choice for selected young SLE patients. 

In addition, Nozu et al. [38] treated five adolescents with SLE MZB 300 mg/day orally in two divided doses, which is twice the conventional dose for adults. Patients 1 and 2 had been treated with prednisolone and CyA, but as the duration of CyA administration became long, it was replaced with 300 mg MZB, and the transition was accomplished smoothly. Patient 3 experienced repeated recurrences during treatment with PSL and CyA or CPM, but the symptoms were controlled by the addition of MZB. In patients 4 and 5, symptom control with prednisolone alone was judged to be difficult, and concomitant treatment with MZB 300 mg was started and enabled a decrease in the dose of prednisolone. The Cmax (C2) of MZB was 1.33 *μ*g/mL or higher in all five patients, and the efficacy of the treatment was satisfactory. Hyperuricemia developed as a side effect in two patients, but it resolved in one of them after reducing the dose of MZB and it resolved spontaneously in the other patient while the treatment was continued. Temporary exacerbation of hair loss was observed in two patients, but it resolved in both of them after a few months. It was possible to administer MZB could be administered at a high dose effectively and safely. However, monitoring of the serum uric acid level was necessary. High-dose MZB therapy showed sufficient efficacy and safety to warrant its application to the treatment of steroid-dependent pediatric patients with SLE.

### 3.5. Other Renal Disease

There had been a few reports on the efficacy of MZB against other renal disease besides IgAN, nephrotic syndrome, and lupus nephritis. Imaizumi et al. [39] reported that steroid pulse and MZB combined with plasmapheresis may be an effective treatment in a patient with focal segmental glomerulosclerosis complicated by CyA-induced leukoencephalopathy. Hirayama et al. [40] investigated the efficacy of MZB in patients at high risk of relapse of ANCA-associated renal vasculitis. Their study was conducted on 5 patients, 4 with myeloperoxidase (MPO) anti-neutrophil cytoplasmic antibody- (ANCA-) associated renal vasculitis and 1 with proteinase 3 (PR3) ANCA-associated renal vasculitis, in whom remission had been achieved by treatment with methylprednisolone pulse therapy, corticosteroids, and cyclophosphamide. MZB therapy was started when their ANCA titers were found to be above the normal range after the remission. The median time between the initial treatment and first dose of MZB was 40.0 months (range: 24–51 months), and the median follow-up period was 13.0 months (range: 6–16 months). Before the start of MZB therapy, none of the patients had experienced a relapse, and their ANCA titers 3 months before the start of MZB therapy were below the limit of detection. When MZB administration was started, the ANCA titers of all of the patients were elevated (median MPO-ANCA, 101 ELISA units (EU); range: 65–154 EU; PR3-ANCA, 55 EU), but no new symptoms or signs of relapse were noted. After 2 months of MZB therapy, only 1 patient had experienced a relapse, but the ANCA titers of all of the other patients had decreased, and in 3 patients they had become normal. Considering the balance between suppression of disease activity and adverse effects of treatment, MZB may be useful as preemptive treatment for patients with ANCA-associated renal vasculitis at high risk of relapse.

### 3.6. Adverse Reactions

Various kinds of clinical trials and a postmarketing surveillance study involved a total of 4906 cases receiving MZB therapy for kidney transplantation and three disease patients. The principal adverse reactions associated with the use of MZB were leucopenia, abnormal hepatic function, rash, increased levels of uric acid, and vomiting. Adverse reactions that occurred at a rate of 0.5% or greater in the patient population for at least one induction of MZB are presented in Table 5 [4].

## 4. Conclusions

The review has summarized the published findings regarding the efficacy of MZB in the treatment of renal disease including IgAN, lupus nephritis and NS, of oral MZB pulse therapy for severe lupus nephritis, and NS, and of the mechanism of the effect oral MZB pulse therapy on the lymphocyte cell cycle. It will, of course, be necessary to further evaluate the efficacy of MZB and oral MZB pulse therapy for the above renal diseases by means of randomized control trials with long-term follow-up before MZB is used worldwide.

## Figures and Tables

**Table 1:** Changes in serum MZB concentration and AVC0-4 in the 11 patients who received oral MZB pulse therapy.

Case	Serum MZB concentration (*μ*g/mL)						AUC0-4 (*μ*g · h/mL)
	C0	C1	C2	C3	C4	C24	
1	0.0	2.8	4.5	2.9	2.1	0.0	12.3
2	0.0	1.4	2.2	4.2	3.4	0.0	11.2
3	0.0	1.8	3.5	4.4	3.9	0.0	13.6
4	0.0	1.7	2.5	3.8	1.9	0.0	9.9
5	0.0	4.1	5.1	3.9	2.9	0.0	16.0
6	0.0	1.2	3.0	4.2	4.9	0.0	13.3
7	0.0	2.2	3.8	3.6	2.5	0.0	12.1
8	0.0	2.2	2.7	3.0	2.5	0.0	10.4
9	0.0	1.4	2.7	3.3	2.4	0.0	9.8
10	0.0	1.4	2.5	2.6	2.0	0.0	8.5
11	0.0	1.6	1.8	2.2	1.4	0.0	7.0

Serum MZB was measured immediately before the morning dose of MZB (C0) and 1 hour (Cl), 2 hours (C2), 3 hours (C3), 4 hours (C4), and 24 hours (C24) after the dose.

AUC = area under the concentration-time curve; MZB = mizoribine.

**Table 2:** Comparison between patient characteristics and laboratory findings in the three groups at the time of the first renal biopsy.

	PWD (*n* = 21)	PWD-pulse (*n* = 20)	PWDM (*n* = 20)	
Age at first renal biopsy, years	11.5 ± 2.9	13.1 ± 4.3	12.9 ± 3.2	n.s.
Time between onset of symptoms and biopsy, months	6.2 ± 6.9	6.0 ± 4.9	5.5 ± 5.1	n.s.
Male:female ratio	11 : 10	12 : 8	11 : 9	n.s.
Urinary protein excretion, mg/m^2^/hr	69 ± 55	72 ± 49	78 ± 68	n.s.
Patients with severe proteinuria (<50 mg/m^2^/hr)	110 ± 79* *(*n* = 12)	99 ± 36* *(*n* = 13)	101 ± 57* *(*n* = 12)	n.s.
Patients with mild proteinuria (<50 mg/m^2^/hr)	36 ± 11* *(*n* = 9)	42 ± 8* *(*n* = 7)	38 ± 12* *(*n* = 8)	n.s.
Hematuria (macroscopic)	21 (13)	20 (14)	20 (12)	n.s.
Serum albumin, g/L	31 ± 4	32 ± 6	30 ± 5	n.s.
Serum creatinine, *μ*mol/L	60 ± 22	64 ± 28	61 ± 25	n.s.
24-hour Ccr, mg/min/1.73 m^2^	84 ± 33	82 ± 35	79 ± 29	n.s.

n.s.: not significant.

**Table 3:** Comparison between laboratory findings in the three groups at the latest follow-up examination.

	PWD (*n* = 21)	PWD-pulse (*n* = 20)	PWDM (*n* = 20)
The duration from initiation of therapy (years)	8.9 ± 5.2	8.1 ± 3.9	7.7 ± 3.8
Urinary protein excretion (mg/m^2^/hr)	17 ± 10^(a)^	22 ± 20^(b)^	6 ± 6^(a,b)^
Patients with severe proteinuria (<50 mg/m^2^/hr)	19 ± 9^(c)^	25 ± 22^(d)^	5 ± 5^(c,d)^
Patients with mild proteinuria (<50 mg/m^2^/hr)	14 ± 8^(e)^	18 ± 15^(f)^	7 ± 6^(e,f)^
Hematuria (macro) (cases)	15 (5)^(g)^	14 (6)^(h)^	5 (0)^(g,h)^
Serum albumin (g/L)	34 ± 4	32 ± 6	36 ± 5
Serum creatinine (*μ*moI/L)	52 ± 39^(e,i)^	78 ± 59^(e,i)^	44 ± 15^(i)^

^(a,c,d,e,f,g,h)^ *P* < .05.

^(b,i)^ *P* < .01.

**Table 4:** Comparison between clinical stages in the three groups at the time of the first renal biopsy and the most recent follow-up examination.

	First renal biopsy			Most recent follow-up examination		
	PWD (*n* = 21)	PWD-pulse (*n* = 20)	PWDM (*n* = 20)	PWD (*n* = 21)	PWD-pulse (*n* = 20)	PWDM (*n* = 20)
Stage 0	0/21 (0%)	0/20 (0%)	0/20 (0%)	2/21 (10%)^(e)^	3/20 (15%)^(f)^	12/20 (60%)^(e,f)^
Stage 1	0/21 (0%)	0/20 (0%)	0/20 (0%)	7/21 (35%)	6/20 (30%)	7/20 (35%)
Stage 2	17/21 (81%)^(a)^	14/20 (70%)^(a,b)^	15/20 (75%)^(b)^	11/21 (52%)^(g)^	8/20 (40%)^(h)^	1/20 (5%)^(g,h)^
Stage 3	4/21 (19%)^(c)^	6/20 (30%)^(c,d)^	5/20 (25%)^(d)^	1/21 (5%)^(i)^	3/20 (15%)^(i,j)^	0/20 (0%)^(j)^

^(e,f,g,h)^ *P* < .05.

^(a,b,c,d,i,j)^n.s.: not significant.

**Table 5:** Adverse reactions to MZB.

	Renal Transplantation	Lupus Nephritis	Rheumatoid Arthritis	Nephrotic Syndrome	Total
Number of surveyed cases	916	275	3478	240	4909
Number of adverse reaction cases	186	33	462	38	719
Number of adverse reaction episodes	229	47	658	49	983
Incidence of adverse reaction cases	20.31%	12.00%	13.28%	15.83%	14.65%

Incidence of Adverse Reactions (%)					

Blood					
Leukopenia	6.99	2.55	0.20	0.83	1.63
Thrombocytopenia	0.98	0.73	0.14		0.33
Anaemia	0.44	0.36	0.72	0.42	0.63
Infection					
Pneumonia	0.55		0.03	0.42	0.14
Mycosis pulmonary	0.55				0.10
Herpes zoster	0.76	1.45	0.17		0.35
Other viral infection	0.76		0.03		0.16
Liver					
Hepatic function abnormal	4.15	1.09	1.18	2.29	1.81
Hypersensitivity					
Rash		1.09	1.64	2.08	1.32
Prurigo			1.09		0.77
Metabolic					
Uric acid increased	1.64	1.45	0.43	2.50	0.81
Gastrointestinal					
Celialgia		0.36	2.21	1.67	1.67
Anorexia	0.98	1.09	1.12	0.83	1.08
Vomiting	0.55	1.09	1.18	0.42	1.02
Nausea	0.11	1.09	0.23		0.24
Diarrhea	0.22	0.73	0.63	0.42	0.55
Stomatitis	0.76	0.73	0.66	0.42	0.67
Skin					
Epilation	1.09	1.09	0.29	1.67	0.55

Note. Listed adverse reactions occurred in 0.5% of cases of greater for at least one indication of MZR.
